# Iron-Based Hollow Nanoplatforms for Cancer Imaging and Theranostics

**DOI:** 10.3390/nano12173023

**Published:** 2022-08-31

**Authors:** Shun Luo, Shuijie Qin, Gerile Oudeng, Li Zhang

**Affiliations:** 1 Key Laboratory for Photoelectronic Technology and Application, Guizhou University, Guiyang 550025, China; 2 Department of Hematology and Oncology, Shenzhen Children’s Hospital, Futian, Shenzhen 518038, China; 3 School of Science, Harbin Institute of Technology (Shenzhen), Shenzhen 518055, China

**Keywords:** iron-based hollow nanoplatforms, theranostics, magnetic resonance imaging, multifunctional

## Abstract

Over the past decade, iron (Fe)-based hollow nanoplatforms (Fe-HNPs) have attracted increasing attention for cancer theranostics, due to their high safety and superior diagnostic/therapeutic features. Specifically, Fe-involved components can serve as magnetic resonance imaging (MRI) contrast agents (CAs) and Fenton-like/photothermal/magnetic hyperthermia (MTH) therapy agents, while the cavities are able to load various small molecules (e.g., fluorescent dyes, chemotherapeutic drugs, photosensitizers, etc.) to allow multifunctional all-in-one theranostics. In this review, the recent advances of Fe-HNPs for cancer imaging and treatment are summarized. Firstly, the use of Fe-HNPs in single T_1_-weighted MRI and T_2_-weighted MRI, T_1_-/T_2_-weighted dual-modal MRI as well as other dual-modal imaging modalities are presented. Secondly, diverse Fe-HNPs, including hollow iron oxide (IO) nanoparticles (NPs), hollow matrix-supported IO NPs, hollow Fe-complex NPs and hollow Prussian blue (PB) NPs are described for MRI-guided therapies. Lastly, the potential clinical obstacles and implications for future research of these hollow Fe-based nanotheranostics are discussed.

## 1. Introduction

Cancer, a group of diseases in which tumor cells proliferate malignantly, is regarded as one of the deadliest threats to human health [1,2,3]. Though chemotherapy, radiotherapy and surgery have been extensively investigated for clinical cancer treatment, insufficient curative outcomes and severe side effects still hamper the further applications of these traditional therapeutic paradigms [4,5,6]. With the rapid advancement of nanomedicine, novel cancer treatment modalities such as phototherapy and catalytic therapy have become more and more popular [7,8,9,10]. Specifically, photothermal therapy (PTT) utilizing different photothermal agents (PTAs) is able to absorb near-infrared (NIR, 700–1700 nm) light and convert the light energy to heat for cancer ablation [11,12]. Chemodynamic therapy (CDT) employs diverse transition metal ions to catalyze endogenous hydrogen peroxide (H_2_O_2_) to generate highly reactive and toxic hydroxyl radicals (•OH) to induce cancer cell death [13,14]. As single-modal therapy is not capable of completely eradicating tumors, nowadays, researchers are focused on the design and fabrication of versatile nanosystems for combinational therapies [15,16,17,18].

An accurate diagnosis of cancer is a prerequisite for efficient treatment. Magnetic resonance imaging (MRI) meeting clinical diagnostic requirements is particularly effective in detecting soft tissues, due to the merits including non-invasive nature, high resolution and penetration depth [19,20,21,22]. In addition to sophisticated instrumentation and imaging methods, MRI depends on the use of contrast agents (CAs) [23,24,25]. Based on the different types of MRI, the CAs can be divided into T_1_ and T_2_ two categories [26]. T_1_ CAs shorten the longitudinal relaxation time of water protons and thus brighten the tumor sites [22,27,28,29,30,31,32], whereas T_2_ CAs shorten the transverse relaxation time of water protons and produce dark signals [33,34,35,36]. Up to now, paramagnetic Gd(III) complexes [37,38,39] and superparamagnetic iron oxide (IO) nanoparticles (NPs) [40,41,42] are the representatives of clinical T_1_ and T_2_ CAs, respectively.

Over the past decade, Fe-based nanomaterials have been of great interest for cancer theranostics, owing to their excellent biocompatibility and biosafety, unique magnetic performance as well as superior therapeutic features [43,44,45]. So far, Fe-based nanomaterials have been widely involved in magnetic hyperthermia therapy (MHT), PTT and CDT, etc. [46,47,48,49]. As for MRI, apart from the feasibility of IO NPs as T_2_-weighted MRI CAs, Fe-based complexes and ultrasmall IO NPs are ideal T_1_-weighted MRI CAs [50,51]. Notably, PTAs utilized for PTT are also ideal photoacoustic imaging (PAI) CAs [52,53]. To enlarge the diagnostic and therapeutic efficacy, Fe-based hollow nanoplatforms (Fe-HNPs) have attracted great attention [54,55,56,57]. On one hand, Fe species themselves, such as magnetic IO NPs, can be ideal hollow nanocarriers [58]. On the other hand, several hollow NPs can serve as the matrices to support different Fe components [59]. Up to now, the synthetic strategies for hollow nanostructures are mainly divided into two categories, i.e., the sacrificial-template-based method (e.g., the utilization of different hard and soft templates) [60,61] and the self-templating method (e.g., the involvement of the nanoscale Kirkendall effect, galvanic replacement reaction and Ostwald ripening process) [62,63]. Of note, Fe-based nanoplatforms not only display great promise in MRI, the cavity of which are also capable of loading diverse fluorescent dyes, chemotherapeutic drugs, photosensitizers for fluorescence imaging (FLI), chemotherapy and photodynamic therapy (PDT), respectively. For example, Ying et al. [64] reported a magnetic nanocatalytic system comprised of glucose oxidase-loaded hollow iron oxide nanocatalysts (HIONCs) to drive starvation–chemodynamic–hyperthermia synergistic cancer therapy. Cai et al. [65] used hollow Prussian blue (PB) NPs to load chemotherapeutic drugs that could achieve efficient chemotherapeutic-thermal treatment of tumors in vivo. In short, multifunctional all-in-one theranostics can be easily obtained with rational and ingenious design on the basis of Fe-HNPs [66,67,68,69].

**Scheme 1:**
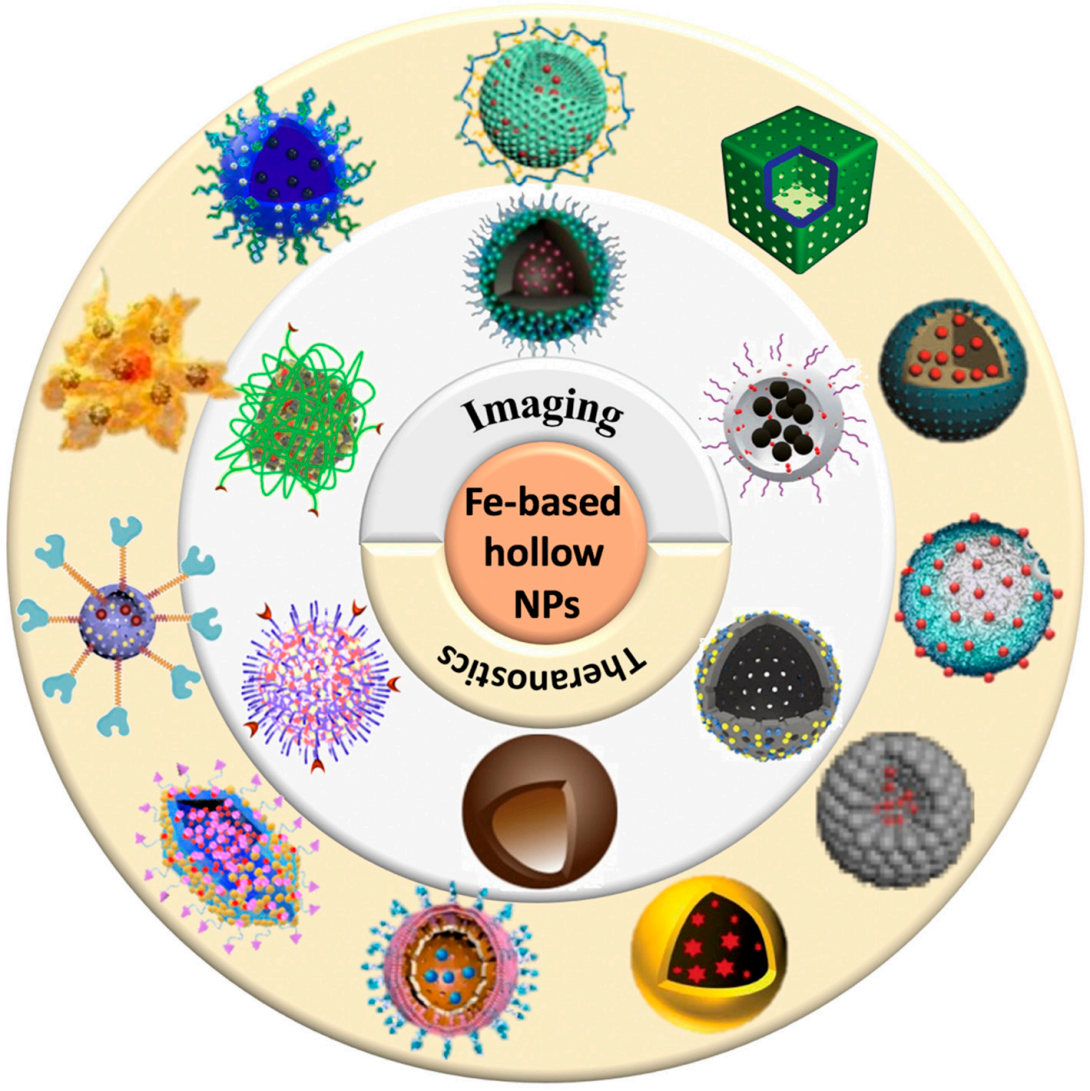
Schematic illustration of various Fe-HNPs for cancer imaging and theranostics.

In this review, we present the recent progress of diverse Fe-HNPs for cancer theranostics (Scheme 1). First of all, the use of Fe-HNPs in single T_1_-weighted MRI and T_2_-weighted MRI, T_1_-/T_2_-weighted dual-modal MRI as well as other dual-modal imaging modalities are summarized. Subsequently, the representative Fe-HNPs including hollow IO NPs, hollow matrix-supported IO NPs, hollow Fe-complex NPs and hollow PB NPs for MRI-guided therapies are introduced. Finally, we discuss the potential clinical obstacles and implications for future research of these hollow Fe-based nanotheranostics.

## 2. Fe-HNPs for Cancer Imaging

### 2.1. Single-Modal Imaging

Since Baddeley et al. [70] first imaged the human body using magnetic resonance technology in 1986, MRI has attracted increased attention in biomedical applications [71,72]. Up to now, MRI has been regarded as the most commonly used clinical diagnostic method for different types of cancer [14]. In order to improve the imaging qualities, so-called CAs are widely involved. So far, Fe-based nanoagents have been proved to be good candidates as T_1_- and T_2_-weighted MRI CAs [51,73,74]. In this section, diverse Fe-HNPs for single-modal T_1_- and T_2_-weighted MRI are presented.

#### 2.1.1. T_1_-Weighted MRI

**Figure 1:**
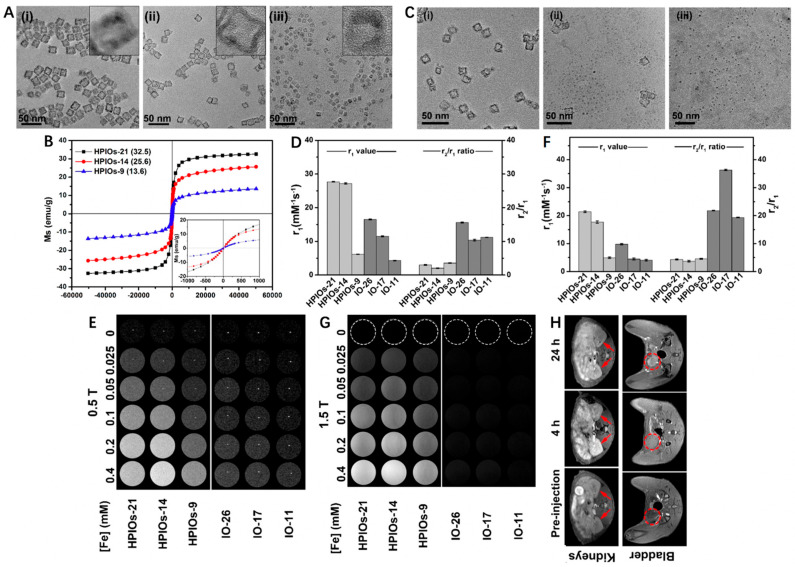
(**A**) Transmission electron microscopy (TEM) images and high-resolution TEM (HRTEM) images (inset) of HPIOs-21 (**i**), HPIOs-14 (**ii**), and HPIOs-9 (**iii**). (**B**) Field-dependent magnetization curves (M−H) of HPIOs-21, HPIOs-14, HPIOs-9 at a magnetic field of 5 T at 300 K (insets: magnification of M−H curves from −1000 to 1000 Oe). (**C**) TEM images of HPIOs-14@ZDS incubated in PBS (pH 7.4) buffer for 1 (**i**), 2 (**ii**), and 4 (**iii**) h, respectively. Relaxation measurements of HPIOs and IOs at (**D**) 0.5 and (**F**) 1.5 T. T_1_-weighted phantom imaging of HPIOs-21, HPIOs-14, HPIOs-9, IO-26, IO-17, and IO-11 on (**E**) 0.5 T and (**G**) 1.5 T MRI scanners. (**H**) In vivo T_1_ MR imaging in the axial plane of rats before and at 4 and 24 h after injection of HPIOs-14 at a dose of 0.05 mmol Fe/kg body weight. Arrows and dotted circles indicate the kidneys and bladder, respectively. Reprinted with permission from Ref. [58]. Copyright 2018, American Chemical Society.

Traditional magnetic IO NPs are typical T_2_ CAs, but their feasibilities for T_1_-weighted MRI have also been investigated in recent years. For example, Wei et al. [58] developed a biodegradable and kidney-cleavable HPIOs@ZDS for enhanced T_1_-weighted MRI. In this work, hollow porous γ-Fe_2_O_3_ nanoboxes (HPIOs) with different side lengths (21 nm for HPIOs-21, 14 nm for HPIOs-14, and 9 nm for HPIOs-9) were first synthesized by redox etching of Mn_3_O_4_ nanocubes using Fe(II) ions, followed by surface modification with amphoteric dopamine sulfonate (ZDS) (Figure 1A). As shown in Figure 1B, all HPIOs exhibited superparamagnetic behavior at 300 K and the saturation magnetizations (MS) of HPIOs decreased with the descending side length. Interestingly, the hollow structure collapsed after incubation of HPIOs-14@ZDS with PBS buffer (pH 7.4) for 1, 2, and 4 h, respectively, indicating its degradable manner at normal physiological environment (Figure 1C). In comparison with IO-26, IO-17 and IO-11 (r_1_ = 16.5, 11.5 and 4.3 mM^−1^ s^−1^, respectively), the HPIOs-21, HPIOs-14 and HPIOs-9 at a 0.5 T scanner separately showed much higher r_1_ values of 27.7, 27.2 and 6.2 mM^−1^ s^−1^. Meanwhile, the HPIOs-21, HPIOs-14, and HPIOs-9 displayed reduced r_2_/r_1_ ratios in contrast to IO-26, IO-17, and IO-11, respectively, suggesting that HPIOs possessed better T_1_-weighted MRI performance than ordinary solid IOs (Figure 1D,E). Similar trends were also noticed using 1.5 T MRI scanners, the improvement of r_1_ values in HPIOs could be explained as the hollow porous structures allowed water molecules to enter the cavity of the HPIOs and coordinate with the internal paramagnetic ion centers (Figure 1F,G). As for in vivo MRI, the T_1_ signal in the kidney was significantly enhanced and peaked at 4 h post injection of HPIOs-14, while the contrast in the bladder continued to strengthen (Figure 1H). These results revealed that HPIOs could be substantially biodegraded and cleared by the kidney in the body. With excellent T_1_ imaging performance, good self-degradation, and renal clearance, such HPIOs-14@ZDS are promising as clinical T_1_ CAs for accurate MRI.

In other work, Liu et al. [75] successfully synthesized hollow ultrasmall Fe_3_O_4_ NPs with diameters of 7 nm and 10 nm by adjusting the reaction temperature during the thermal decomposition of pentacarbonyl iron (Fe(CO)_5_). Similarly, ZDS exhibiting excellent water dispersibility and permeability was encapsulated on hollow Fe_3_O_4_ NPs to allow highly passive tumor targeting capacity. Compared with the 10-Fe_3_O_4_@ZDS NPs (r_2_/r_1_ ratio = 18.69), the 7-Fe_3_O_4_@ZDS NPs showed much lower r_2_/r_1_ ratio of 4.0, indicating its superior T_1_-weighted MRI performance. An obvious T_1_ signal was observed after injection of the 7-Fe_3_O_4_@ZDS NPs (40 mg kg^−1^) for 60 min, and reached the maximum at 180 min with a 23% signal enhancement. Besides, the 7-Fe_3_O_4_@ZDS NPs also proved to be efficiently excreted by the kidneys.

#### 2.1.2. T_2_-Weighted MRI

As for T_2_-weighted MRI, Cheng et al. [76] used SiO_2_ NPs as the hard template to prepare bilayer shell-structured Fe_3_O_4_@C nanocapsules (HMNPs). The amorphous carbon layer outside of the Fe_3_O_4_ NPs endowed the nanoplatforms with good water-dispersibility, enhanced stability and reduced toxicity. Specifically, over 80% of the cells survived when the HMNPs concentration reached up to 1000 mg/L. The capability of HMNPs as ideal CAs were further investigated on a 3.0 T MR system. As expected, the signal enhanced with the increase in Fe concentration and the r_2_ was determined to be 54.55 mM^−1^ s^−1^ for HMNPs.

Xu et al. [77] prepared hollow magnetic iron oxide nanoclusters (HMNC) for enhanced T_2_-weighted MRI based on Gd-doped magnetic iron oxide nanoclusters (MIONC). MIONC had a flower-like structure with a particle size of 80~100 nm, while the HMNC displayed a morphology in hollow ring and its diameter was 150~200 nm. These changes in size and structure indicated that the Gd doping led to grain rearrangement in MIONC and thus formed HMNC. As revealed by the field-dependent magnetization curves, the MIONC had a typical saturated magnetization value (42.5 emu/g) at 300 K with no remanence and coercive force found, but the magnetic properties of HMNC were significantly different. Subsequently, the r_2_ values of the MIONCs before and after Gd doping were calculated to be 294.1 and 449 mM^−1^ s^−1^ at 3.0 T and 372.4 and 669.2 mM^−1^ s^−1^ at 7.0 T, respectively, suggesting that the doping of Gd could facilitate the contrast enhancement of T_2_ MRI.

**Figure 2:**
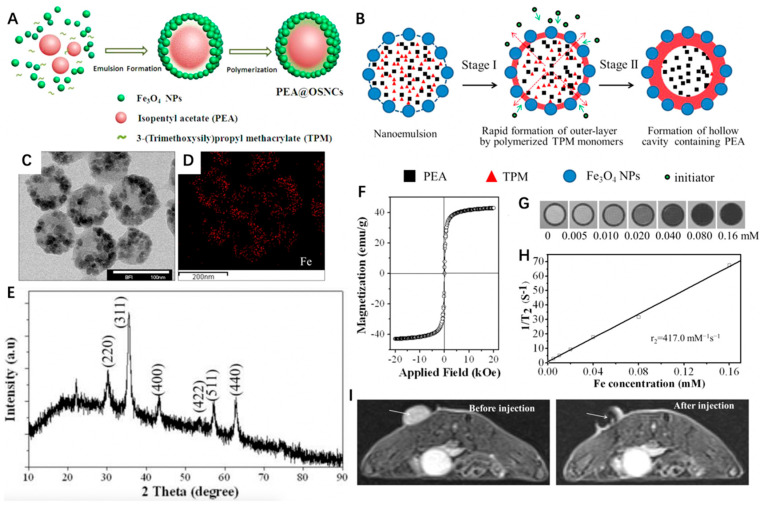
(**A**) Schematic illustration of the synthesis of PeA@OSNC. (**B**) Schematic illustration of the structural conversion process from nanoemulsion to hollow structure. (**C**) TEM images of PeA@OSNC. (**D**) Fe element mapping corresponding to panel B, displaying the distribution of Fe_3_O_4_ nanoparticles in the overall structure. (**E**) XRD pattern of PeA@OSNC presents the characteristic diffraction peaks of magnetite (Fe_3_O_4_) crystals including (220), (311), (400), (511), (422) and (440) lattice planes. (**F**) Field-dependent magnetization curve of OSNC at room temperature. Absence of hysteresis loop confirms the superparamagnetism of our prepared sample. (**G**) T_2_-weighted MR phantom images and (**H**) plot of inverse transverse relationship times (1/T_2_) versus Fe concentration for OSNC. (**I**) In vivo MR imaging of tumor-bearing mice before and after intratumoral injection of PeA@OSNC suspension. Arrows point to tumor tissue. Reprinted with permission from Ref. [78]. Copyright 2016, American Chemical Society.

Through a facile and template-free synthetic strategy, Ma et al. [78] constructed a unique hollow core/satellite shell-structured organosilica nanocapsule (OSNC) with Fe_3_O_4_ embedded onto the shell and isopentyl acetate (PeA) liquid encapsulated in the cavity (denoted as PeA@OSNC) (Figure 2A). The structural transformation of Fe_3_O_4_ NPs stabilized by 3-(trimethoxysilyl)propyl methacrylate (TPM)/PeA oil nanoemulsion to the final hollow structure of OSNC could be divided into two stages. First, the polymerized organosilica outer layer embedded with numerous Fe_3_O_4_ NPs could be formed immediately upon addition of the initiator potassium persulfate (KPS), because the outermost TPMs of the nanoemulsion were earlier involved in radical polymerization compared to the interior ones. Afterward, the polymerization continuously occurred in the sealed liquid phase where the free TPMs tended to gradually diffuse outward from the center and took part in shell formation, thus leaving a cavity containing PeA (Figure 2B). As we can see from Figure 2C–E, the PeA@OSNC NPs exhibited a uniform spherical structure with an average diameter of 82 nm, in which the uniformly distributed small NPs (<10 nm) could be ascribed to Fe_3_O_4_ NPs. According to the field-dependent magnetization curve, the MS value of OSNC was 42.9 emu g^−1^ (Figure 2F). The feasibility of OSNC as a negative CA for MRI was then investigated, the contrast of images showed a gradual decrease with the increased Fe concentration, and the corresponding r_2_ was calculated to be 417.0 mM^−1^ s^−1^ (Figure 2G,H). As for in vivo MRI, the mean signal intensity of the tumor site displayed a significant decrease from 188 to 52 after injection of PeA@OSNC for 1 h. These results indicated the great promise of as-synthesized PeA@OSNC for future clinical application in cancer diagnosis and drug delivery systems.

### 2.2. Dual-Modal Imaging

**Scheme 2:**
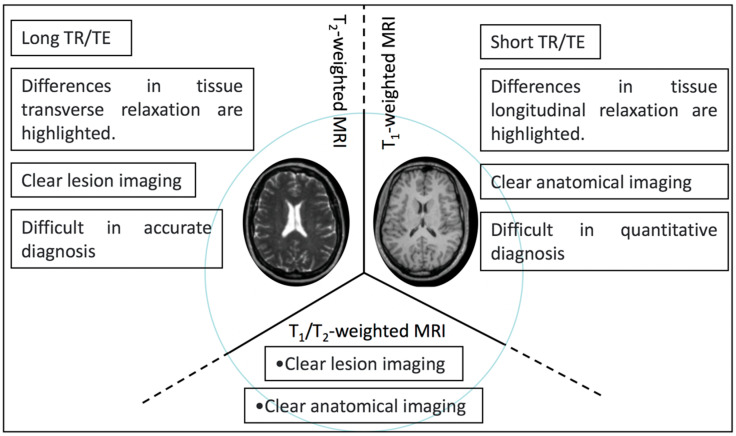
Schematic illustration of the characteristics of T_1_-, T_2_-, and T_1_/T_2_-weighted dual-modal MRI.

Nevertheless, T_1_- or T_2_-weighted MRI alone cannot achieve accurate clinical diagnosis, multi-modal imaging has become a research hotspot over the past decade as it can combine the advantages of each imaging method [26]. By combing two kinds of magnetic cores, T_1_-T_2_ dual-modal MRI with exceptional accuracy and reliable manner has received considerable attention over the past years [79,80] (Scheme 2).

**Figure 3:**
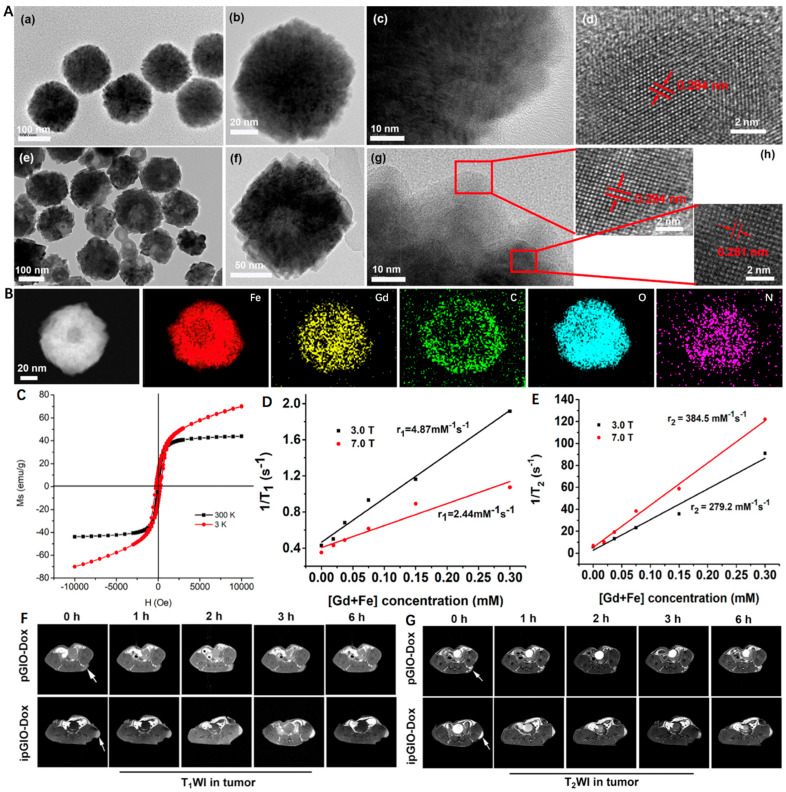
(**A**) TEM images of (**a**–**c**) MIO and (**e**–**g**) GdIO. HRTEM images of (**d**) MIO and (**h**) GdIO. (**B**) Energy dispersive spectrometry mapping images of GdIO. (**C**) Field-dependent magnetization curve of GdIO at 3 and 300 K. (**D**) Transverse relaxation rate and (**E**) Longitudinal relaxation rate of ipGdIO-Dox measured using 3.0 T and 7.0 T MRI scanners. (**F**,**G**) T_1_- and T_2_-weighted MR images of cancer-bearing mice at coronal planes after injection of pGdIO-Dox and ipGdIO-Dox. Reprinted with permission from Ref. [81]. Copyright 2022, American Chemical Society.

For example, Zhu et al. [81] developed a novel magnetic nanoplatform (denoted as GdIO) that consisted of Gd species and MIO for T_1_ and T_2_ dual-modal MRI. Briefly, Fe(acac)_3_ and Gd(acac)_3_ were first selected as precursors and underwent a pyrolysis process, followed by surface modification with iRGD-PEG-ss-PEG (denoted as ipGdIO) to improve the colloidal stability and endow active targeting ability. As shown in Figure 3A, the MIO exhibited typical spherical morphology with an average diameter of ~150 nm, while an obvious hollow cavity was noticed in GdIO after Gd engineering. Moreover, the two kinds of interplanar spacing at 0.294 nm and 0.281 nm could be ascribed to the (220) plane of the spinel structure of magnetite and (302) plane of Gd(OH)CO_3_, respectively. Correspondingly, the element mapping images displayed the even distribution of Fe, Gd, C, O, and N, indicating the successful preparation of GdIO (Figure 3B). Moreover, the GdIO with excellent superparamagnetism possessed a higher saturation magnetization intensity of 42.5 emu/g at 300 K. Due to the existence of Gd, the magnetization of GdIO showed gradual enhancement with the increase of magnetic field but could not reach saturation at 3K (Figure 3C). According to Figure 3D,E, the r_1_ of ipGdIO at 3.0 T and 7.0 T were calculated to be 4.87 and 2.44 mM^−1^ s^−1^, while the r_2_ were 279.2 and 384.5 mM^−1^ s^−1^, respectively. As for in vivo MRI experiments, the nanocrystals with iRGD functionalized showed brighter T_1_-weighted images of tumors when compared to the non-targeted group. The signal-to-noise ratio change (ΔSNR) reached over 65% occurring at 3 h, which was about two times higher than that of the non-targeted one (Figure 3F). At the same time, the maximum T_2_-weighted images in the targeted group was 1.5 times that of the iRGD-free group (Figure 3G). This work provides a new route for constructing Fe/Gd nanoplatform as T_1_ and T_2_ dual-modal MRI Cas.

In addition, Huang et al. [82] decorated Fe_3_O_4_/Gd_2_O_3_ hybrid NPs on the surface of the hollow mesoporous organosilica nanoparticles (HMONs), displaying excellent T_1_/T_2_ dual-modal MRI properties owing to the superparamagnetic nature of Fe_3_O_4_ (r_1_ = 20.1 mM^−1^ s^−1^) and paramagnetic performance of Gd^3+^ (r_2_ = 253.7 mM^−1^ s^−1^). Analogously, Zhang et al. [83] utilized hollow carbon nanospheres (HCSs) to carry GdPO_4_ and γ-Fe_2_O_3_ for T_1_- and T_2_-weighted MRI (r_1_ = 3.78 mM^−1^ s^−1^ and r_2_ = 257.48 mM^−1^ s^−1^). In addition to Fe-Gd nanosystems, Sun et al. [84] synthesized a biodegradable Fe-Mn nanoplatform (denoted as MnSiO_3_@Fe_3_O_4_) to enable T_1_/T_2_ dual-modal MRI, in which r_1_ and r_2_ were calculated to be 12.24 and 66.62 mM^−1^ s^−1^, respectively, at acidic glutathione (GSH) solution (pH = 5.5, GSH = 10 μM).

Highlighting the differences in T_1_ longitudinal relaxation between tissues, T_1_-weighted magnetic resonance imaging helps researchers to observe anatomical structures. Highlighting the differences in T_2_ transverse relaxation between tissues, T_2_-weighted magnetic resonance imaging is suitable for watching tissue lesions. Compared with T_1_- and T_2_-weighted MRI, T_1_/T_2_-weighted dual-mode MRI has the advantages of both imaging types, giving it better imaging performance, and the imaging characteristics between them are shown in Scheme 2.

Apart from T_1_-T_2_ dual-modal MRI, other imaging modalities have also been reported to integrate with MRI to compensate for the defects of each other [85]. FLI features high imaging sensitivity, and for the first time, Wang et al. [86] stably and uniformly loaded upconversion nanoparticles (UCNPs) onto magnetic carbon-based nanocomposites (FeCUPs) to enable MRI as well as FLI. Moreover, as an emerging diagnostic technique which integrates the advantages of both optical and ultrasound imaging, PAI has attracted increasing attention in recent years [87,88]. Through a simple coating-etching strategy, Su et al. [89] developed a nanoplatform (denoted as Ag-HPB) on the basis of hollow PB nanocubes with Ag nanoparticles well dispersed, displaying great promise for dual-modal MRI/PAI. In addition, Chen et al. [90] utilized a co-precipitation method to synthesize ultra-small Fe_3_O_4_ cores within the cavity of ZrO_2_ NPs for dual-modal MRI/CT imaging.

## 3. Fe-HNPs for MRI-Guided Cancer Therapies

Although some achievements have been made in cancer imaging, the ultimate goal is the cure of cancer. Theranostics show tremendous potential for delivering precision medicine that integrate both diagnostic and therapeutic functions in one single nanoplatform [91,92]. In addition to MRI, Fe-based NPs also can initiate diverse cancer therapies including MHT, PTT and CDT, etc. Moreover, hollow structures are able to further enlarge the imaging and treatment efficacy as the cavities can be used to encapsulate various small molecules such as fluorescent dyes, chemotherapeutic drugs, photosensitizers for FLI, chemotherapy and PDT, respectively. In this section, we introduce four main categories of Fe-HNPs including hollow IO NPs, hollow matrix-supported IO NPs, hollow Fe-complex NPs and hollow PB NPs, and their biomedical applications as inspiring cancer nanotheranostics are also summarized.

### 3.1. Hollow IO-Based Nanoplatforms

Up to now, the most commonly investigated Fe-mediated cancer therapy paradigm is CDT, which can be activated by endogenous stimulus and shows high selectivity [93,94]. However, the treatment efficacy of CDT is still hampered by the mild pH, limited H_2_O_2_ level and overproduced GSH [95]. To this end, several methods have been reported accordingly to improve CDT, such as the decrease in intratumoral pH and GSH concentration as well as the enhancement of H_2_O_2_ level. Moreover, the combination of other therapeutic strategies is also regarded as a potent way to boost CDT efficiency [96].

**Figure 4:**
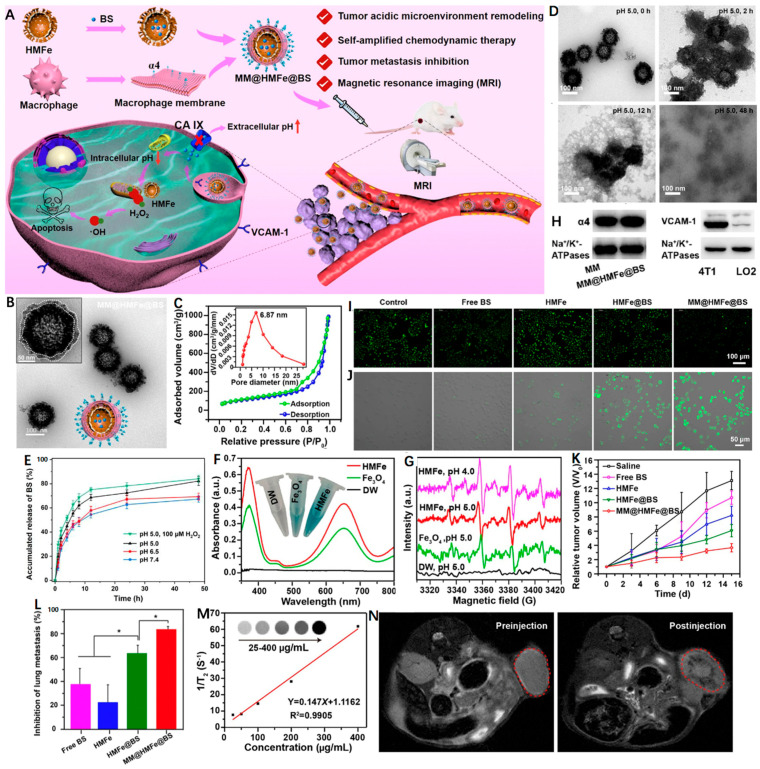
(**A**) Illustration of MM@HMFe@BS for self-amplified chemodynamic therapy and tumor metastasis inhibition via tumor microenvironment remodeling under the monitoring of MRI. (**B**) Representative TEM images of MM@HMFe@BS. (**C**) Nitrogen adsorption−desorption curve of the HMFe. Inset shows the pore size of HMFe. (**D**) Time-dependent decomposition behavior of MM@ HMFe@BS. (**E**) In vitro release profiles of BS from MM@HMFe@BS at various pH values (5.0, 6.5, or 7.4) with or without H_2_O_2_. (**F**) UV−Vis absorption spectra of TMB after coincubation with DW (deionized water), solid Fe_3_O_4_, and HMFe in the presence of H_2_O_2_ (100 μM) at pH 5.0. (**G**) ESR spectra of detected •OH at different conditions. (**H**) Western blot analysis of α4 and VCAM-1 in macrophage membrane and 4T1 cells membrane, respectively. Fluorescence images of (**I**) BCECF-stained and (**J**) APF-stained 4T1 cells treated with free BS, HMFe, HMFe@BS, and MM@HMFe@BS for 12 h. (**K**) Relative tumor volume changes in 4T1 tumor-bearing mice with different treatments (n = 5). (**L**) Lung metastasis inhibition ratios on the 15th day post-treated with different formulations in contrast to control group (n = 5). (**M**) Linear plot of 1/T_2_ as a function of HMFe concentration. The inset is T_2_-weighted MRI images of HMFe. (**N**) In vivo MRI of 4T1 tumor-bearing mice before and after intravenous injection of MM@HMFe@BS. Reprinted with permission from Ref. [99]. Copyright 2022, American Chemical Society. * *p* < 0.05.

Carbonic anhydrase IX (CA IX) as a pH-regulating enzyme is overexpressed in tumors that can lower the extracellular pH to promote the extracellular matrix degradation and accelerate tumor metastasis [97]. Interestingly, 4-(2-aminoethyl)benzenesulfonamide (BS) is a CA IX inhibitor (CAI) that is able to effectively inhibit tumor metastasis as well as induce intracellular H^+^ accumulation [98]. In light of this, Zuo et al. [99] reported a macrophage-mimic hollow mesoporous Fe-based nanocatalysts with BS loaded (denoted as MM@HMFe@BS) to remodel the TME for self-amplified CDT and metastasis inhibition under MRI guidance (Figure 4A). The monodisperse MM@HMFe@BS possessed an obvious virus-like hollow mesoporous shape and an average particle size of ~148 nm (Figure 4B). The high specific surface area (374.44 m^2^ g^−1^) and pore size (∼6.87 nm) allowed the HMFe to encapsulate BS with a loading efficiency of 15.8 ± 4.9 wt % (Figure 4C). The macrophage membrane (MM) modification proved to prevent the premature BS leakage at normal physiological environment (pH 7.4), but for acidic conditions (pH 5.0), the MM@HMFe@BS gradually collapsed and complete degradation of the virus-like hollow mesoporous structure was noticed after 48 h (Figure 4D). Accordingly, the releasing behavior of BS from MM@HMFe@BS was investigated at different pH values, and of note, H_2_O_2_ treatment could further boost the BS release at pH 5.0 (Figure 4E). This process may be ascribed to the generated •OH from the HMFe-induced Fenton reaction. As shown in Figure 4F,G, the intensive blue color, characteristic absorption peak (~652 nm) and typical electron spin resonance (ESR) signals (1:2:2:1) confirmed the production of •OH (Figure 4F,G). Figure 4H revealed that the MM@HMFe@BS was selectively uptaken by 4T1 cells via α4 integrin/VCAM-1 interaction. Next, by utilizing BCECF-AM as a pH probe, the TME-remolding capability of BS was reflected by the decreased green fluorescence intensity and the stronger intracellular acidity resulted in more •OH formation (Figure 4I,J). As expected, the MM@HMFe@BS-treated groups showed the most significant tumor suppression due to the prolonged blood circulation and prominent tumor accumulation effect together with self-amplified CDT efficiency (Figure 4K). More importantly, the MM@HMFe@BS displayed remarkable antimetastatic activity against breast cancer (Figure 4L). In addition, the feasibility of MM@HMFe@BS as a T_2_-weighted MRI CA was also demonstrated (Figure 4M,N). This study provides a new paradigm for designing more effective CDT strategy against metastatic breast cancer.

In other work, the macrophage-mediated nanoplatform has also been reported in diagnosing and treating glioma [100]. Briefly, hollow porous Fe_3_O_4_ magnetic NPs were first synthesized to carry Cy5.5, followed by incubation with primary macrophages. The as-prepared Fe_3_O_4_-Cy5.5-loaded macrophages (denoted as MFe_3_O_4_-Cy5.5) possessing targeting ability was able to cross the blood−brain barrier (BBB) and accumulate into deep gliomas. With desirable probing depth and high signal-to-noise ratio, Fe_3_O_4_-Cy5.5 could perform triple-modal FLI/PAI/MRI to distinguish brain tumors from the surrounding healthy tissues and precisely guide the resection of glioma. Meanwhile, the local hyperthermia generated from PTT was capable of effectively inhibiting the tumor recurrence. Taken together, such a simply designed nanoplatform showed great potential application value for brain tumor theranostics.

**Figure 5:**
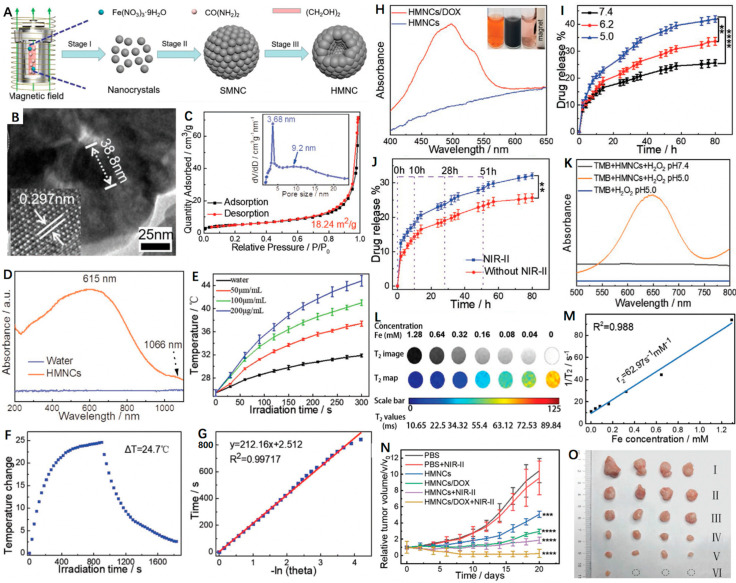
(**A**) Schematic representation of the formation of magnetic-induced HMNCs. **(B**) HRTEM image of single HMNCs; the lower lattice of the HMNCs is a single magnetite stripe. (**C**) N_2_ adsorption–desorption isotherm of the as-synthesized HMNCs, the inset is the pore size distribution of the HMNCs. (**D**) UV–Vis–NIR absorption spectra of water (control) and the HMNCs aqueous solution. (**E**) Temperature change in pure water (control) and HMNCs solution at different concentrations under NIR-II irradiation (1064 nm, 0.8 W cm^−2^) for 300 s. (**F**) Temperature change in HMNCs solution (200 µg mL^−1^) under NIR-II irradiation time (1064 nm, 0.8 W cm^−2^); radiation was stopped after 900 s. (**G**) The relationship between the linear time data obtained from the cooling time shown in Figure 4F and -lnθ. (**H**) UV–Vis absorption spectra of the HMNCs and the DOX-loaded HMNCs aqueous solution; the inset: (left) pure DOX solution, (middle) DOX solution mixed with the HMNCs, and (right) the supernatant after magnetic separation. (**I**) Responsive release of DOX from the DOX-loaded HMNCs over time at various pH values (7.4, 6.0, and 5.0) in a 37 °C solution. (**J**) Responsive release of DOX from the DOX-loaded HMNCs with or without NIR-II irradiation over different time points (1064 nm, 0.8 W cm^−2^, 5 min) in PBS solution at pH = 7.4 and 37 °C. (**K**) Vis–NIR absorption spectra of TMB (oxTMB) treated by the HMNCs and H_2_O_2_ at different pH values. (**L**) T_2_weighted MR images and quantitative T_2_ maps of the HMNCs at different concentrations. (**M**) Plot of 1/T_2_ (R_2_) as a function of iron concentration in the HMNCs. (**N**) Tumor growth curve of mice treated by different conditions (PBS, I; PBS plus NIR-II, II; HMNCs, III; DOX-loaded HMNCs, IV; HMNCs plus NIR-II, V; DOX-loaded HMNCs plus NIR-II, VI). (**O**) Typical photos of the tumors collected from mice with different treatments at the end of the treatment (day 20). Reprinted with permission from Ref. [106]. Copyright 2021, Wiley-VCH GmbH. ** *p* < 0.01, *** *p* < 0.001, **** *p* < 0.0001.

PTT has been demonstrated to improve CDT as the thermal effect is capable of accelerating the Fenton processes [101,102]. Specifically, the Fenton reaction rate can be increased 4-fold when heating an area from 20 to 50 °C [103]. Nowadays, researchers are focused on designing and fabricating diverse NIR-II (1000–1700 nm) PTAs, as NIR-II PTT displays deeper tumor penetration and higher maximum permissible exposure (MPE) (1 W cm^−2^ for 1064 nm, 0.72 W cm^−2^ for 980 nm and 0.33 W cm^−2^ for 808 nm) and thus results in less tissue attenuation in contrast to NIR-I PTT [104,105]. Additionally, hollow nanoplatforms prove to be excellent drug delivery systems that can response to diverse stimuli such as pH and NIR light to release the loaded cargoes on demand. Of note, doxorubicin (DOX) is most widely explored among the reported model drugs. Recently, Wang et al. [106] constructed a novel hollow magnetite nanocluster (HMNC) to load DOX for MRI-guided NIR-II PTT/CDT/chemotherapy (Figure 5A). As shown in Figure 5B,C, the as-prepared HMNC was composed of ~38.8 nm nanocrystals, displaying a hollow spherical shape with an average particle size of 215 ± 20 nm and pore size of 3.68 nm, respectively. Moreover, the HMNCs possessed intensive optical absorption in the NIR-II region, indicating its potential as a good NIR-II PTA (Figure 5D). Upon a 1064 nm laser irradiation, the temperature of the HMNCs dispersions showed obvious enhancement with time (Figure 5E). According to the previous method, the photothermal conversion efficiency (PCE) of HMNCs was calculated to be 36.3% (Figure 5F,G). Moreover, the mesoporous structure and hollow cavity allowed the HMNCs to transport small molecule drugs. Figure 5H presented the characteristic absorption peak around 477 nm, indicating the successful loading of DOX. Of note, both low pH and NIR laser irradiation were able to accelerate the release rate of DOX (Figure 5I,J). These could have contributed to the degradation of the HMNCs and increase in water solubility of DOX as well as the destruction of the electrostatic interaction between DOX and the HMNCs. Furthermore, the chemodynamic ability of HMNCs was investigated that could generate •OH in acidic H_2_O_2_ solution to convert colorless tetramethylbenzidine (TMB) to blue oxidized-TMB (Figure 5K). In addition, due to the superior ferromagnetic performance, the HMNCs exhibited good MRI capability with a calculated r_2_ of 62.97 mM^−1^ s^−1^ (Figure 5L,M). As for the therapeutic effects, in vivo experiments showed that tumor growth was almost completely inhibited in the DOX-HMNCs + NIR-II group of mice because of the synergistic chemotherapy/PTT/CDT (Figure 5N,O). In short, the HMNCs as a versatile nanoplatform provides great promise for targeted imaging-guided tri-modal cancer therapy.

### 3.2. Hollow Matrix-Supported IO Nanoplatforms

In addition to the hollow magnetic IO NPs, Fe-components have also been integrated with other hollow matrices. Therefore, the diagnostic and therapeutic performances of IO NPs together with the intrinsic features of different matrices can realize a greater breakthrough in cancer theranostics. Among the reported hollow matrices, carbon-based nanomaterials are more superior as they often show good photothermal effects for PTT, resulting in improved treatment outcomes when cooperating with CDT originated from the IO NPs.

For example, Wu et al. [59] reported a GSH, pH and NIR light triple stimuli-responsive nanoplatform based on magnetic hollow and porous carbon nanoparticles (MHPCNs) for cancer-specific MRI-guided synergistic photothermal/chemotherapy. In this design, SiO_2_ as a sacrificial template was first used to absorb ferrocene, followed by H_2_O_2_ treatment to prepare SiO_2_@Fe_3_O_4_@C NPs. After removal of SiO_2_ core, the carboxylic groups of obtained MHPCNs were further covalently conjugated with cystamine dihydrochloride to synthesize MHPCNs–SS. Subsequently, pH-sensitive poly(γ-glutamic acid) (PGA) was employed to block the pores with DOX loaded and folic acid (FA) was modified to endow the MHPCNs–SS–PGA/DOX with targeting ability. When the MHPCNs–SS–PGA–FA/DOX turned into cancer cells via the endocytosis process, the PGA layer was detached owing to the low pH and high GSH level, leading to the DOX release for chemotherapy. Moreover, IO NPs and the carbon layer proved to be decent PTAs, which converted the NIR light into heat for tumor ablation. Meanwhile, the releasing rate of DOX could be promoted upon laser irradiation. Such synergetic manner enabled the MHPCNs–SS–PGA–FA/DOX to realize significant tumor suppression with low side effects.

**Figure 6:**
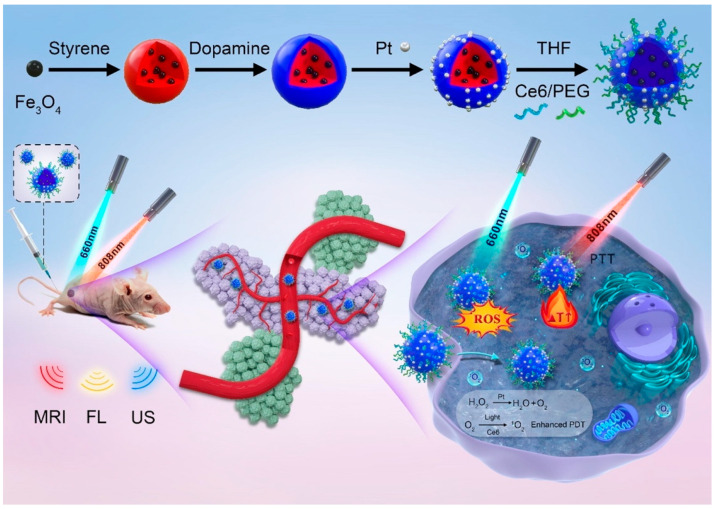
Schematic illustration of the preparation of yolk-shell Fe_3_O_4_@PDA@Pt-PEG-Ce6 and the use of the new nanoplatform for imaging and tumor phototherapy. Reprinted with permission from Ref. [107]. Copyright 2022, Elsevier Inc.

Yan et al. [107] reported a multifunctional yolk-shell nanoplatform (denoted as Fe_3_O_4_@PDA@Pt-PEG-Ce6) for multi-modal imaging-guided synergistic PDT/PTT (Figure 6). First of all, Fe_3_O_4_ NPs were encapsulated inside polystyrene (PS) NPs via polymerizing styrene in the presence of Fe_3_O_4_ magnetofluid. Subsequently, the resultant Fe_3_O_4_@PS were coated with polydopamine (PDA) that facilitated the in situ generation of platinum (Pt) NPs. After removing the PS template, Ce6 and PEG were consecutively functionalized to obtain the final Fe_3_O_4_@PDA@Pt-PEG-Ce6. The working principles of this nanoplatform were summarized as follows: (1) The Pt NPs were able to catalyze endogenous H_2_O_2_ to produce abundant O_2_ to relieve tumor hypoxia for enhanced PDT; (2) The generated O_2_ bubbles were capable of improving the echogenicity signal of yolk-shell microspheres for solid tumor ultrasonic (US) imaging; (3) Owing to the good photothermal performance of the PDA layer, the Fe_3_O_4_@PDA@Pt-PEG-Ce6 could realize synergistic PTT/PDT when exposed to both 808 nm and 660 nm lasers; (4) The Fe_3_O_4_ and Ce6 were also ideal MRI and FLI CAs that could be used for in vivo PDT and PTT guidance.

**Figure 7:**
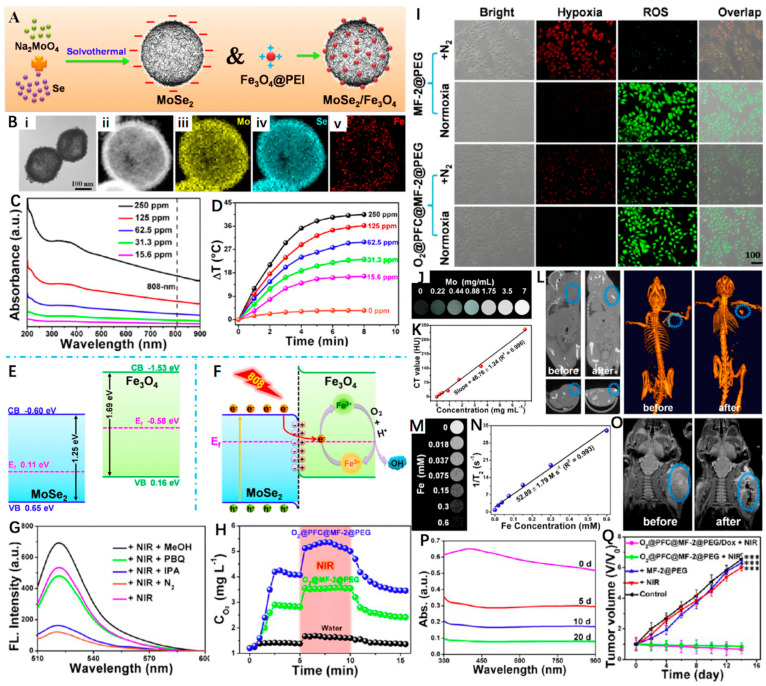
(**A**) Schematic representation of MoSe_2_ and MoSe_2_/Fe_3_O_4_ nanomaterials. (**B**) TEM image of MF-2 and HAADF−STEM image of MF-2 and the corresponding STEM−EDX elemental mapping images for Se, Mo, and Fe. (**C**) UV−Vis spectrum of MF-2 with different concentrations. (**D**) Photothermal heating curves of MF-2 dispersions with different concentrations under an 808 nm laser (1 W cm^−2^) irradiation. (**E**,**F**) Schematic illustration of the energy band configuration of MoSe_2_ and Fe_3_O_4_ and possible mechanism of the charge separation of the MoSe_2_/Fe_3_O_4_ system. (**G**) Fluorescence spectra (excitation at 495 nm) of corresponding sample supernatants (300 μg mL^−1^) and DCFH-DA after irradiation by a NIR laser for 10 min (808 nm, 1 W cm^−2^) under different conditions. (**H**) O_2_ concentration changes after the addition of O_2_@MF-2@PEG or O_2_@PFC@MF-2@PEG into deoxygenated water under without and with 808 nm laser irradiation. (**I**) Fluorescence images of the cells stained with ROS and hypoxia probes. (**J**,**K**) In vitro CT images and relative CT values of MF-2 solution vs. different Mo concentrations. (**L**) CT images of a tumor-bearing mouse before and after injection in situ. (**M**,**N**) T_2_-weighted MR images and corresponding relaxation rates (r_2_) of MF-2 recorded using a 9.4 T MR scanner. (**O**) MR images for in vivo mapping before and after injection of MF-2@PEG. (**P**) Degradation time-dependent UV−Vis spectra of MF-2 in PBS. (**Q**) Changes in the relative tumor volume achieved after various treatments. Reprinted with permission from Ref. [108]. Copyright 2019, American Chemical Society. *** *p* < 0.001.

Besides the carbon-based matrices, other hollow-structured PTAs have also been reported to support IO NPs. For example, Wang et al. [108] decorated polyethyleneimine (PEI)-modified Fe_3_O_4_ onto size- and shell thickness-adjustable hollow MoSe_2_ nanospheres to fabricate a nanoheterostructured nanoplatform (MoSe_2_/Fe_3_O_4_, denoted as MF-2), which was further functionalized with PEG and then served as an ideal nanosystem to load DOX and oxygen carrier perfluorocarbons (PFC) (O_2_@PFC@MF-2@PEG/Dox). As shown in Figure 7A,B, MF-2 possessed an obvious hollow structure and an average diameter and shell thickness of 150 nm and 20 nm, respectively. Moreover, the narrow bandgap of MoSe_2_ and the novel hollow nanoheterostructure endowed the MF-2 with strong NIR absorption, ensuring its excellent photothermal effect with a high PCE of 66.2% at 808 nm (Figure 7C,D). At the meanwhile, the internal electric field at the heterointerface also facilitated the transfer of photogenerated electrons in conduction band (CB) of MoSe_2_ to Fe_3_O_4_. Once trapped by the Fe^3+^, they were reduced to form Fe^2+^ and further reacted with the dissolved oxygen to generate •OH and Fe^3+^ (Figure 7E,F). In addition, Figure 7G revealed that the major ROS type was •OH as the DCF emission was distinctly decreased after isopropanol (IPA, •OH radical scavenger) addition. As an O_2_ carrier, the photothermal effect of MF-2@PEG was able to stimulate the release of O_2_ (Figure 7H). The O_2_@PFC@MF-2@PEG proved to overcome hypoxia and could produce considerable ROS even under N_2_ atmosphere (Figure 7I). Owing to the high X-ray absorption of molybdenum, the superparamagnetic properties of Fe_3_O_4_, the MF-2@PEG were demonstrated to be good CAs for MRI and CT imaging (Figure 7J–O). Through synergistic therapeutic effect of PTT/PDT/chemotherapy, the O_2_@PFC@MF-2@PEG/Dox + NIR group displayed the most significant tumor inhibition rate compared to the other treatment groups (Figure 7Q). More interestingly, the acidity-induced degradation behavior was also evidenced that ensured the biosafety of the as-synthesized hollow nanoplatform (Figure 7P). Based on these, the O_2_@PFC@MF-2@PEG/Dox was expected to play a vital role in potential clinical applications.

### 3.3. Hollow Fe-Complex-Based Nanoplatforms

Owing to the merits of pH response, good biocompatibility and minimal side effects, the coordination polymer Fe–gallic acid (Fe–GA) has been regarded as one of the most promising drug carriers [109]. For the first time, Liu et al. [110] reported hollow Fe–GA nanospheres with the help of bovine serum albumin (BSA) under mild reaction conditions, which could encapsulate DOX for MRI-guided synergistic chemotherapy/CDT/PTT. Briefly, the as-synthesized hollow Fe–GA/BSA@DOX was able to be effectively degraded in TME and release Fe(III) ions as well as loaded DOX. Moreover, the Fe(III) was capable of depleting the overexpressed GSH in cancer cells to generate Fe(II) to facilitate the Fenton reaction. Furthermore, the Fe–GA/BSA@DOX was found to be a good PTA (PCE = 67.14%) that rapidly converted NIR light into heat. In addition, in vitro and in vivo MRI studies confirmed the T_1_ and T_2_ dual-modal imaging performances of Fe–GA/BSA, demonstrating its great potential for synergistic cancer MRI and chemotherapy, CDT, and PTT.

**Figure 8:**
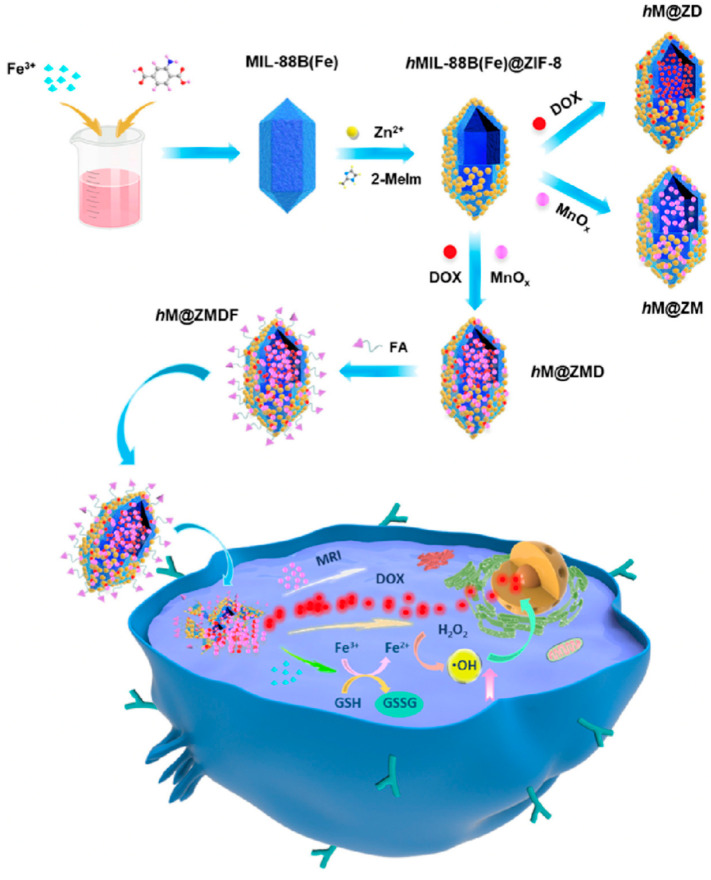
Schematic fabrication routes of hM@ZMDF nanoplatforms based on MIL-88B(Fe) and their applications in cancer-targeted combined therapy. Reprinted with permission from Ref. [113]. Copyright 2019, American Chemical Society.

Metal−organic frameworks (MOFs) brought about widespread attention in the biomedical fields, especially acting as versatile nanotheranostic by incorporating chemotherapeutic drugs, imaging agents, and targeting units [111,112]. Zeng et al. [113] reported a facile method to fabricate a novel hollow and monodisperse MOF (denoted hMIL-88B(Fe)@ZIF-8), in which the spindle-like MIL-88B(Fe) NPs were partially etched by 2-methylimidazole with the assistance of Zn^2+^ to form the cavity. The obtained hMIL-88B(Fe)@ZIF-8 was then used to load DOX, manganese oxide (MnO_x_) NPs, and folic acid (FA) to prepare a multifunctional nanoplatform (denoted hM@ZMDF) (Figure 8). As expected, the resultant hM@ZMDF displayed specific targeting abilities towards MCF-7 and HepG-2 cells with FA receptor overexpressed. Once entered into the cancer cells, the hM@ZMDF showed rapid responsiveness to the TME, resulting in the release of DOX and Fe^3+^. Of note, DOX played dual roles as a chemotherapeutic drug as well as an FLI CA, while the Fe^3+^ could trigger the Fenton reaction and intracellularly generate •OH in the presence of high GSH level. Additionally, the MRI capability of the hM@ZMDF was also investigated, revealing its great potential for smart cancer theranostics.

Conventional mesoporous silica NPs have been extensively explored as ideal drug delivery nanosystems for decades, but their nondegradable manners still hamper the further applications [114,115]. In recent years, it has been found that metal ions-doped organosilicon NPs show rapid responsiveness towards TME and can serve as biodegradable nanocarriers [116,117]. Zhou et al. [118] prepared an Fe-doped hollow mesoporous organosilicon (HMON) transferrin (Tf)-modified and DOX-loaded nanoplatform (denoted as DOX@Fe-HMON-Tf) for MRI-guided enhanced ferroptosis/chemical combination therapy of cancer. In this design, the anti-tumor efficacy was achieved through sequential TME-activatable reactions. Specifically, the wide cavities of Fe-HMON were beneficial to effectively load DOX, which served as both a chemotherapeutic agent and a H_2_O_2_ generator. Surface modification with transferrin enabled the nanoplatform to target the overexpressed transferrin receptors in hepatocellular carcinoma (HCC) cells and facilitate tumor accumulation as well as intracellular DOX delivery. In the presence of upregulated GSH in TME, the Fe-HMON was disintegrated, leading to rapid DOX release, enhanced tumor inhibition efficacy, reduced toxic effects, and improved tolerance. Moreover, the Fe^2+^/Fe^3+^ Fenton reaction, and H_2_O_2_ supply by DOX could induce HCC cell ferroptosis. This mechanism reflected coordinated chemotherapy and CDT cascade. Furthermore, the Fe-HMON-Tf were superparamagnetic, and the feasibility of which as T_2_-MRI CA was demonstrated to guide and monitor therapeutic process. In short, such a multifunctional nanoplatform was able to reverse drug resistance, inhibit HCC recurrence and metastasis, and integrate accurate diagnosis, efficacious treatment, as well as real-time monitoring, showing great potential in clinical cancer nanomedicine.

### 3.4. Hollow PB-Based Nanoplatforms

PB approved by USA Food and Drug Administration (FDA) has been utilized for treating radioactive exposure in clinic [119,120]. With numerous merits including hollow mesoporous structure, effective Fe ions active sites, decent optical absorption and PCE in the NIR area, hollow mesoporous Prussian blue nanoparticles (HMPBs) are expected to be an excellent drug carrier and PTA for enhanced cancer therapy [45,121]. For example, Cai et al. [122] reported a novel core–shell hollow-structured nanoplatform (denoted as HMPB-Mn) by introducing Mn-containing PB analogue (MnPBA) to both the outer surface and the inner mesoporous channels of HMPB. Owing to the hollow mesoporous structure and high surface area, the as-synthesized HMPB-Mn could serve as an ideal nanocarrier for DOX with high loading efficiency (97.5%) and loading capacity (62.3%). Notably, its highly pH-sensitive manner enabled both Mn^2+^ ions and DOX to be released in TME, and the Mn^2+^-induced enhancement of MRI was in favor of real-time monitoring of the DOX release rate. More importantly, through combining the chemotherapy with intrinsic photothermal effect, HMPB-Mn displayed a great potential in synergistic tumor treatment.

**Figure 9:**
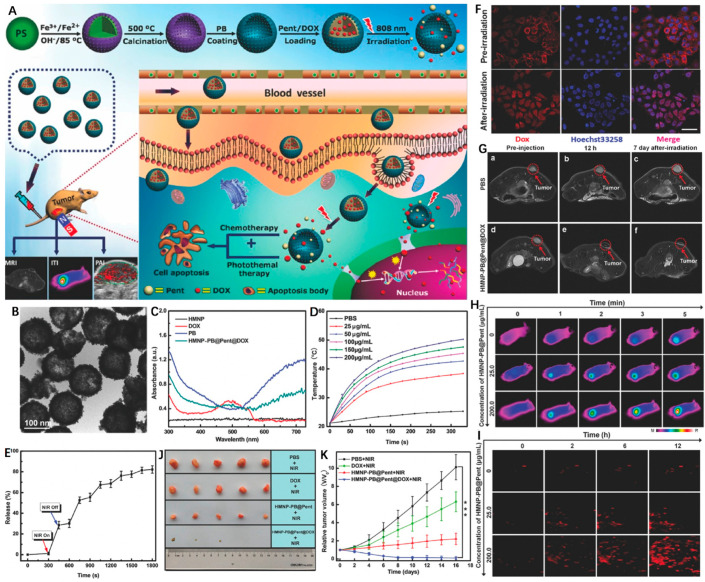
(**A**) Schematic illustration of the design and synthesis of HMNP-PB@Pent@DOX for NIR-guided synergetic chemo-photothermal tumor therapy with tri-modal imaging in vivo. (**B**) TEM image of HMNP-PB. (**C**) Representative UV–Vis–NIR absorption spectra of HMNP, DOX, PB, and HMNP-PB@Pent@DOX NPs in PBS. (**D**) Concentration-dependent thermogenesis of HMNP-PB@Pent@DOX in PBS irradiated by NIR laser (808 nm, 1.2 W cm^−2^, 5 min). (**E**) The on–off switch drug release of HMNP-PB@Pent@DOX NPs in PBS with NIR laser (808 nm, 1.2 W cm^−2^). (**F**) CLSM images of HepG2 cells incubated with HMNP-PB@Pent@DOX NPs before and after irradiation by NIR laser (808 nm, 1.2 W cm^−2^) for 5 min. (**G**) T_2_-MR images of tumors of (**a**) preinjection of PBS, (**b**) after injection of PBS for 12 h, (**c**) after injection of PBS for 7 d with NIR laser irradiation, (**d**) preinjection of HMNP-PB@Pent@DOX, (**e**) after injection of HMNP-PB@Pent@DOX for 12 h, and (**f**) after injection of HMNP-PB@Pent@DOX for 7 d with NIR laser irradiation. (**H**) Infrared thermographic images of tumor-bearing nude mice irradiated with NIR laser. (**I**) PAI signals in the tumors before and after injection of HMNP-PB@Pent@DOX for 2, 6, and 12 h. (**J**) Photographs of the solid tumors after different treatments for 16 d. (**K**) Relative tumor volumes of mice with different treatments. Reprinted with permission from Ref. [125]. Copyright 2017, Wiley-VCH GmbH. *** *p* < 0.001.

In recent years, phase-change materials (PCMs) possessing large latent heats of enthalpy change to realize solid–liquid or liquid–solid phase transformation under certain temperatures have been widely employed in drug delivery systems [123,124]. By coating an FDA-approved PCM (1-pentadecanol, denoted as Pent) on hollow magnetic Prussian blue nanoparticles (HMNP-PB), Li et al. [125] designed and fabricated a “pent-up” drug delivery carrier. Briefly, the HMNP-PB was first constructed by decorating PB onto hollow IO NPs, followed by simultaneous incorporation with chemotherapeutic agent (DOX) and Pent (HMNP-PB@Pent@DOX) for combined MRI/PAI/thermal imaging and PTT/chemotherapy (Figure 9A). The as-synthesized HMNP-PB exhibited regular round morphology with an average diameter of ~131.5 nm (Figure 9B). As shown in Figure 9C, the typical absorption peak around 490 nm (green line) was ascribed to DOX, while the intensive NIR absorption indicated the feasibility of HMNP-PB as good photothermal agent. Under NIR laser irradiation, the HMNP-PB@Pent@DOX dispersion displayed a significant temperature increment (~28 °C) when concentration was 200 μg/mL (Figure 9D). Interestingly, the coated Pent showed rapid response to laser irradiation that was converted from a solid phase into a liquid phase (>42 °C), promoting the release of loaded DOX (Figure 9E). The vivid red fluorescence confirmed the efficient cellular uptake of HMNP-PB@Pent@DOX, which was transferred from the cytoplasm to nuclei with 5 min laser irradiation due to the accelerated DOX release (Figure 9F). As reflected in the MRI, thermal imaging and PAI images, the HMNP-PB@Pent@DOX showed high tumor accumulation after systemic administration according to the EPR effect (Figure 9G–I). On account of the synergistic effect of chemo-photothermal treatment, the HMNP-PB@Pent@DOX achieved superior in vivo therapeutic outcomes in contrast to chemotherapy or PTT alone (Figure 9J,K).

## 4. Conclusions and Perspectives

Over the past years, hollow nanoplatforms especially Fe-HNPs prove to be excellent candidates for cancer nanotheranostics. This work presents recent progress of Fe-HNPs and their applications in cancer imaging such as single-modal T_1_- or T_2_-weighted MRI and MRI-based dual-modal imaging, as well as MRI-guided cancer therapies that were summarized according to the materials types including hollow IO-based NPs, hollow matrix-supported IO NPs, hollow Fe-complex-based NPs, and hollow PB-based NPs. In addition to their inherent diagnostic and therapeutic features, the Fe-based hollow nanosystems can also be optimally designed for synergistic cancer imaging and treatment with other units integrated. For better understanding, the above-mentioned Fe-HNPs were summarized in terms of materials, particle sizes, templates and mechanisms, biomedical applications (Table 1). Despite the inspiring advances, several issues are remained to be resolved before the clinical use of those Fe-HNPs. First of all, safety should be taken into consideration when comes to the clinically translational nanomedicine. Although Fe-based components have been demonstrated to possess low toxicity, biocompatibility and biodegradability, the introduced hollow substances may cause risks in biotoxicity. Moreover, more attention should be focused on the surface functionalization of the Fe-HNPs to improve their biocompatibility, physiological safety, blood circulation time and tumor accumulation. Furthermore, the synergetic manners should be explored when integrating various imaging and treatment modalities into one single Fe-based hollow nanoplatform. Additionally, the particle sizes and cavity volumes of the hollow nanoplatforms should be rationally tuned to realize higher tumor accumulation as well as drug loading efficiency. Additionally, last but not least, a simple and mild synthetic strategy for the large-scale production of such novel hollow NPs should be developed. We believe that with future endeavors from different research areas, Fe-HNPs will hopefully translate from the laboratory to the clinic.

## Figures and Tables

**Table 1:** Summary of various Fe-HNPs for cancer imaging and theranostics.

Material	Size (nm)	Templates and Mechanisms	Biomedical Applications	Reference
HPIOs-14@ZDS	14	Mn_3_O_4_	T_1_-weighted MRI	[58]
7-Fe_3_O_4_@ZDS	7	–	T_1_-weighted MRI	[75]
HMNPs	120	SiO_2_	T_2_-weighted MRI	[76]
PeA@OSNC	82	–	T_2_-weighted MRI	[78]
HMNC	150−200	Self-assembly	T_2_-weighted MRI	[77]
ipGdIO	160	–	T_1_-/T_2_-weighted MRI	[81]
HMON2@FG3	47.8	SiO_2_	T_1_-/T_2_-weighted MRI	[82]
Gd–Fe/HCSs	100	SiO_2_	T_1_-/T_2_-weighted MRI	[83]
MnSiO_3_@Fe_3_O_4_	198	SiO_2_	T_1_-/T_2_-weighted MRI	[84]
FeCUPs	216	–	T_2_-weighted MRI/FLI	[86]
Ag-HPB	69	Acid etching	T_1_-weighted MRI/PAI	[89]
Fe_3_O_4_@ZrO_2_	155	SiO_2_	T_2_-weighted MRI/CT imaging	[90]
MM@HMFe@BS	148	SiO_2_	T_2_-weighted MRI and CDT/metastasis inhibition	[99]
MFe_3_O_4_-Cy5.5	190	–	T_2_-weighted MRI/PAI/FLI and Surgery/PTT	[100]
DOX-HMNCs	215 ± 20	Ostwald ripening	T_2_-weighted MRI and PTT/CDT/chemotherapy	[106]
MHPCNs-SS-PGA-FA/DOX	127	SiO_2_	T_2_-weighted MRI and PTT/chemotherapy	[59]
Fe_3_O_4_@PDA@Pt-PEG-Ce6	160	PS	T_2_-weighted MRI/FLI/USI and PDT/PTT	[107]
O_2_@PFC@MF-2@PEG/Dox	150	Self-assembly	T_2_-weighted MRI/CT imaging and chemotherapy/PDT/PTT	[108]
Fe-Ga/BSA@DOX	200	–	T_1_-/T_2_-weighted MRI and chemotherapy/PTT/CDT	[110]
hM@ZMDF	102 ± 13	spindle-like MIL-88B(Fe) NPs	T_2_-weighted MRI/FLI and chemotherapy/CDT	[113]
DOX@Fe-HMON-Tf	71	SiO_2_	T_2_-weighted MRI and ferroptosis/chemotherapy	[118]
HMPB-Mn-DOX	290	Acid etching	T_1_-weighted MRI and PTT/chemotherapy	[122]
HMNP-PB@Pent@DOX	131.5	PS	T_2_-weighted MRI/PAI and chemotherapy/PTT	[125]

## Data Availability

Not applicable.
